# Major vault protein supports glioblastoma survival and migration by upregulating the EGFR/PI3K signalling axis

**DOI:** 10.18632/oncotarget.1264

**Published:** 2013-09-01

**Authors:** Daniela Lötsch, Elisabeth Steiner, Klaus Holzmann, Sabine Spiegl-Kreinecker, Christine Pirker, Juraj Hlavaty, Helga Petznek, Balazs Hegedus, Tamas Garay, Thomas Mohr, Wolfgang Sommergruber, Michael Grusch, Walter Berger

**Affiliations:** ^1^ Institute of Cancer Research, Department of Medicine I, Medical University Vienna, Austria; ^2^ Comprehensive Cancer Center-Central Nervous System Tumours Unit, Medical University of Vienna; ^3^ Department of Neurosurgery, Wagner-Jauregg Hospital, Linz, Austria; ^4^ Institute of Virology, Department of Pathobiology, University of Veterinary Medicine Vienna; ^5^ Division of Thoracic Surgery, Department of Surgery, Medical University of Vienna; ^6^ 2nd Institute of Pathology, Semmelweis University, Budapest, Hungary; ^7^ Boehringer Ingelheim RCV GmbH & Co KG, Department of Lead Discovery, Vienna, Austria

**Keywords:** major vault protein, glioblastoma multiforme, invasion, EGFR, PI3K

## Abstract

Despite their ubiquitous expression and high conservation during evolution, precise cellular functions of vault ribonucleoparticles, mainly built of multiple major vault protein (MVP) copies, are still enigmatic. With regard to cancer, vaults were shown to be upregulated during drug resistance development as well as malignant transformation and progression. Such in a previous study we demonstrated that human astrocytic brain tumours including glioblastoma are generally high in vault levels while MVP expression in normal brain is comparably low. However a direct contribution to the malignant phenotype in general and that of glioblastoma in particular has not been established so far. Thus we address the questions whether MVP itself has a pro-tumorigenic function in glioblastoma. Based on a large tissue collection, we re-confirm strong MVP expression in gliomas as compared to healthy brain. Further, the impact of MVP on human glioblastoma aggressiveness was analysed by using gene transfection, siRNA knock-down and dominant-negative genetic approaches. Our results demonstrate that MVP/vaults significantly support migratory and invasive competence as well as starvation resistance of glioma cells *in vitro* and *in vivo*. The enhanced aggressiveness was based on MVP-mediated stabilization of the epidermal growth factor receptor (EGFR)/phosphatidyl-inositol-3-kinase (PI3K) signalling axis. Consequently, MVP overexpression resulted in enhanced growth and brain invasion in human glioblastoma xenograft models. Our study demonstrates, for the first time, that vaults have a tumour-promoting potential by stabilizing EGFR/PI3K-mediated migration and survival pathways in human glioblastoma.

## INTRODUCTION

Human glioblastoma multiforme (WHO grade IV, GBM) represents the most common and aggressive form of primary brain tumour in adults with a median survival of less than 15 months [1]. This dismal prognosis is based on both, the resistance to current therapeutic options [2] and the invasion of tumour cells into the adjacent tissue making total surgical resection mostly unachievable [3]. The successful chemotherapeutic treatment of brain tumours is mainly limited by the blood-brain-barrier and the expression of ABC-transporter proteins, acting as drug efflux pumps [4]. Another protein frequently linked with drug resistance is the major vault protein (MVP), also known as lung resistance protein (LRP) [5]. MVP forms the outer shell of the vault, a large and highly abundant ribonucleoparticle complex [6]. In addition to MVP, vaults contain vault poly(ADP-ribose)-polymerase (vPARP), telomerase-associated protein (TEP-1) and several small untranslated RNAs (vRNA) [7-9]. Multiple *in vitro* studies demonstrated that MVP is almost generally overexpressed in drug-resistant human cancer cells selected against diverse chemotherapeutic agents [10]. However, the role of MVP and vaults in drug resistance is controversially discussed [5, 11, 12].

Vaults are widely expressed in eukaryotic organisms including humans but surprisingly are missing e.g. in flies, worms and plants [9]. Due to their hollow-barrel structure which can dynamically open and close, vaults were suggested to be involved in transport mechanisms [5, 7, 13]. Consequently, vaults are of interest in nanotechnology and are currently developed as natural nano-capsules e.g. for drug delivery applications [14]. Furthermore, vaults participate in the regulation and fine-tuning of a variety of intracellular signal pathways, including mitogen-activated protein kinase (MAPK) and phosphatidylinositol 3-kinase (PI3K) signalling [9]. Additionally, our group has identified MVP as an interferon-stimulated gene regulating phosphorylation and hence nuclear translocation of STAT1 [15].

In the healthy human organism the highest levels of vaults are found in tissues potentially exposed to exo- or endotoxins like the epithelia of the lung and the gastrointestinal tract as well as in macrophages [16]. During malignant transformation or cancer progression MVP expression is initiated or upregulated in various tumours [9] including gliomas [17]. Nevertheless a definite tumour-promoting function of vaults has not been conclusively worked out so far.

Previously, our group and others have reported constitutive upregulation of vaults in cells and tissues of astrocytic brain tumours [17-19]. Consequently, we investigated in this study the impact of MVP overexpression on growth dynamics and aggressiveness of human GBM *in vitro* and *in vivo*. Our data assign -for the first time- a tumour-promoting function to vault particles overexpressed in many cancer types [5].

## RESULTS

### MVP-(over)expression in human GBM

**Figure 1:**
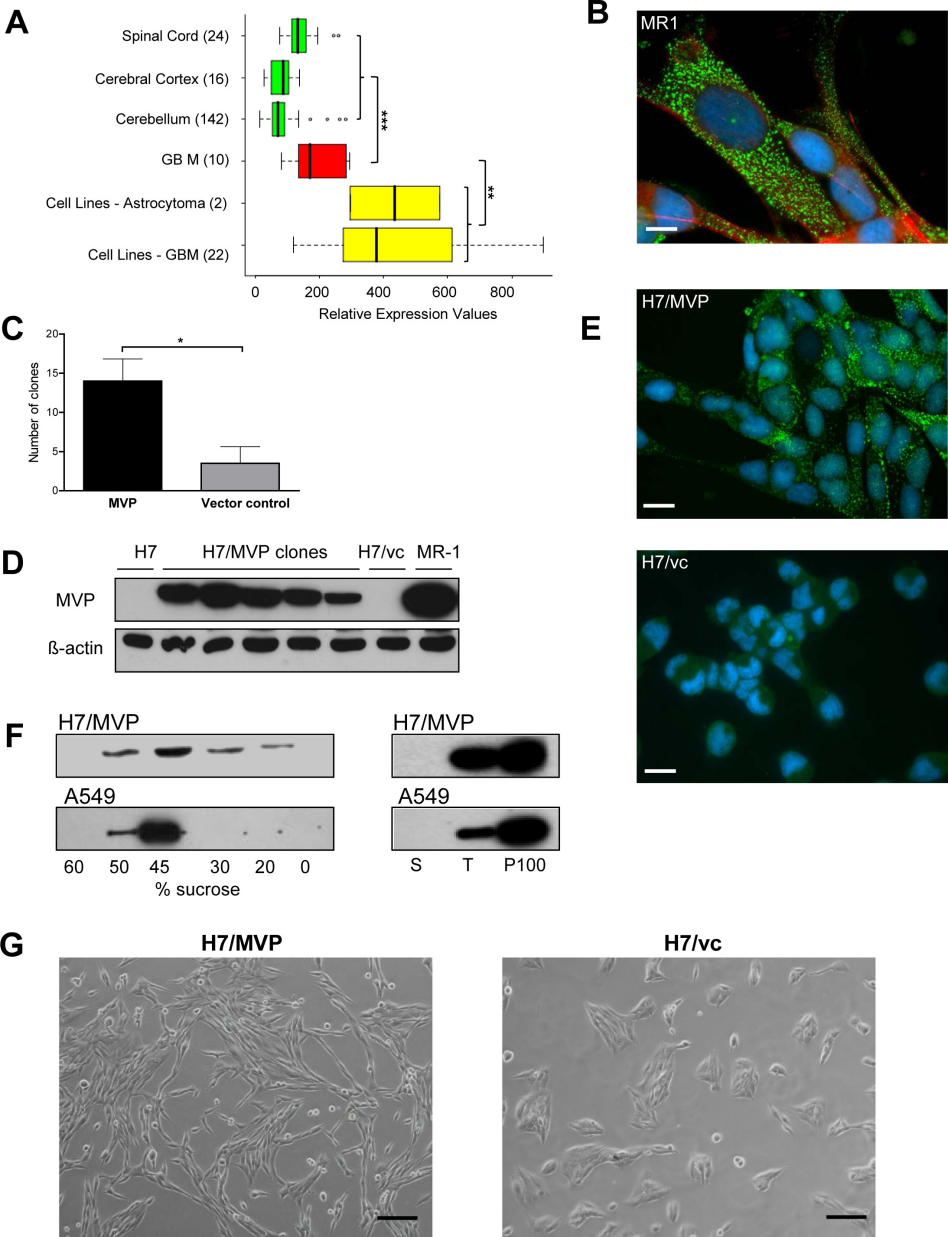
MVP expression in GBM and establishment of stable MVP-overexpressing H7 sublines A. MVP mRNA expression data in normal (green) and malignant (red) brain tissues as well as tumour cell cultures (yellow), determined by gene expression array analyses, were extracted from the BioExpress database (GeneLogic). Sample sizes are given in brackets (Student's t-test). B. Immunofluorescence staining of endogenous MVP (green) in the cytoplasm of MR-1 GBM cells; nuclei (blue) by DAPI; microfilaments (red) by TRITC-phalloidin (bar = 10 μm). C. Clone formation of H7 cells transfected with MVP compared to vector control plasmid was monitored in three experiments (Student's t-test). D. MVP expression of MVP-transfected cell clones as compared to parental H7 and vector control (vc) cells was analyzed by immunoblot. MR-1 GBM cell extracts served as positive control. E. Exogenously expressed MVP (green) was detected by immunofluorescence staining in H7/MVP cells (upper panel) and vector control cells (H7/vc) (lower panel) (scale bar = 20 μm). F. Vault particle formation by exogenous and endogenous MVP was confirmed in H7/MVP and A549 cells [5050], respectively. Sucrose gradient fractions (left panel) and precipitated proteins after 100000×g centrifugation (right panel), were tested for MVP expression by immunoblot. S (supernatant), T (total cell lysate), P100 (100000g pellet). G. Photographs depicting the morphology of MVP-overexpressing H7/MVP and the corresponding vector control (H7/vc) cells (scale bar = 100 μm).

Previously we have reported that cell cultures derived from an extended panel of patients with GBM almost generally overexpress MVP [17]. In the present study we re-confirmed strong MVP gene expression in GBM surgical specimens and especially cell cultures, whereas the healthy cortex, spinal cord and cerebellum were comparably low in MVP mRNA (Figure 1A). Consistent data regarding MVP mRNA expression upregulation in human GBM were derived from diverse *in silico* datasets like https://www.genevestigator.com/gv/ (data not shown). Immunofluorescence staining of MVP revealed a dotted, cytoplasmic distribution pattern indicating formation of vault particles in human GBM cells (shown representatively in the MVP-positive GBM cell line MR-1 in Figure 1B). Out of all patient-derived primary cultures and cell lines analysed, only one GBM cell model, namely H7, almost completely lacked MVP. Consequently we established stable MVP-overexpressing (H7/MVP) and corresponding empty vector control subclones (H7/vc) to analyse the impact on GBM cell behaviour. Selection for MVP-positive clones was significantly more efficient as compared to the vector control already indicating a positive impact of MVP expression on H7 clonogenic cell survival (Figure 1C and D). Comparably to the endogenous MVP, also ectopically expressed MVP localized mainly to dotted, preferentially cytoplasmic structures (Figure 1E). Vault particle formation in H7/MVP cells was also confirmed by 1) 100000×g centrifugation leading to complete MVP pelleting and 2) accumulation of MVP to the 45% fraction in sucrose-gradient centrifugation [10] (Figure 1F). All MVP-positive subclones displayed distinctly changed spindle-shaped morphology as compared to parental and vector control-transfected H7 cells (Figure 1G).

### MVP supports the migratory potential of GBM cells

**Figure 2:**
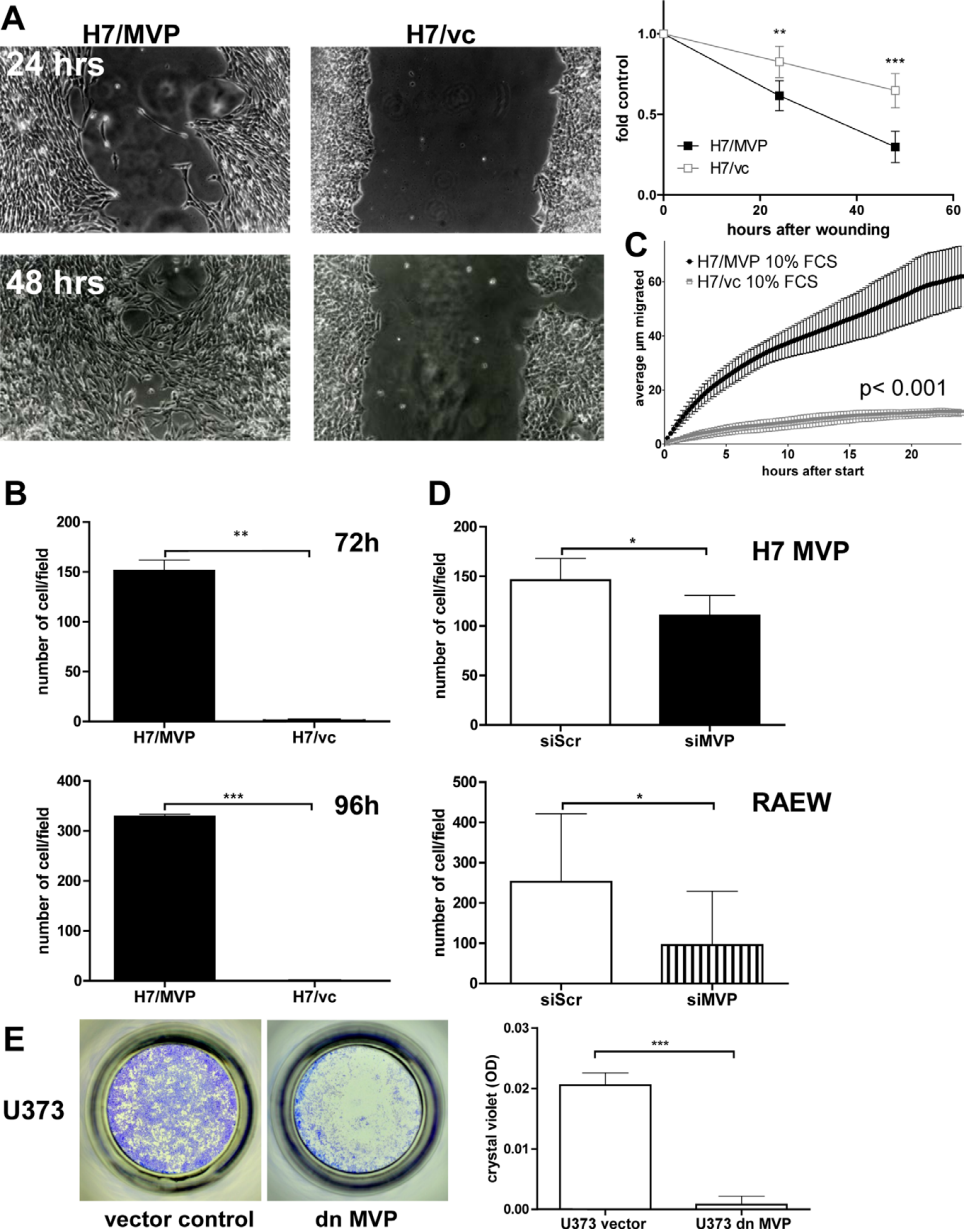
MVP enhances the migratory potential of human GBM cells A. Migratory potentials of H7/MVP and H7/vc cells were analysed by wound healing assays. Quantitative results (right panel) represent the mean ± SD open area at the indicated time points relative to the starting gap set as 1. Three positions of each scratch from two independent experiments were analysed (two-way ANOVA and Bonferroni post-test). B. In transwell-migration assays cells at the bottom of filters were stained with crystal violet after the indicated time points and counted microscopically in 4 optical fields per filter. Bars represent the means ± SD of three independent experiments performed in duplicates (Student's t-test). C. The spontaneous migratory pattern of H7/MVP and H7/vc cells on culture plastic was analysed by videomicroscopy for 24 hours and the average migration distance in the given time interval is shown (two-way ANOVA and Bonferroni post-test). D. The impact of siRNA-mediated reduction of ectopic (H7/MVP) and endogenous (RAEW) MVP on transwell filter migration was determined 96 hours after transfection (compare B, Student's t-test). E. U373 cells were infected with an adenovirus encoding dominant-negative MVP (dnMVP, MOI 10). The impact on transwell filter migration within 72 hours was determined by measuring the absorbance of crystal violet (OD) as described in Material and Methods. D. and E. Bars represent the means ± SD of three independent experiments performed in duplicates (Student's t-test).

Wound-healing assays demonstrated that ectopic MVP expression significantly increased cell migration at all time-points analysed (Figure 2A). This MVP-supported migration was even more pronounced in transwell-chamber experiments (Figure 2B) where only MVP-overexpressing cells were able to cross the pores of the filter. Accordingly, videomicroscopy revealed a robust migratory activity of H7/MVP cells and only minor cell displacements of the vector controls (Figure 2C; Supplementary Movies 1 and 2). In order to confirm that the migratory potential was indeed mediated by vaults, siRNA experiments were performed in MVP-transfected and endogenously MVP-expressing GBM cell models. MVP-targeting siRNA reduced MVP expression at the mRNA (Supplementary Figure S1A) and protein (compare Figure 4C) levels by around 50-60%. Higher efficacy was unobtainable owing to the extended half-life of MVP assembled into vaults [15]. However, already this partial knock-down resulted in a significant reversal of the migratory phenotype in MVP-transfected H7 cells (Figure 2D) while vector control cells remained widely migration-incompetent (Supplementary Figure S1B). Accordingly, MVP siRNA significantly attenuated transwell migration in several endogenously MVP-expressing GBM cell models (RAEW representatively in Figure 2D). In order to reduce the amount of intact vault particles more efficiently, a dominant-negative EGFP-MVP deletion variant (dnMVP) was expressed in GBM cells (compare Material and Methods; Supplementary Figure S2A). Also dnMVP distinctly reduced the migratory potential of MVP-positive GBM cell models as compared to the empty vector control (Figure 2E) again suggesting an important contribution of vaults to the migratory potential of GBM cells.

### MVP supports clonogenicity and starvation resistance of GBM cells

**Figure 3:**
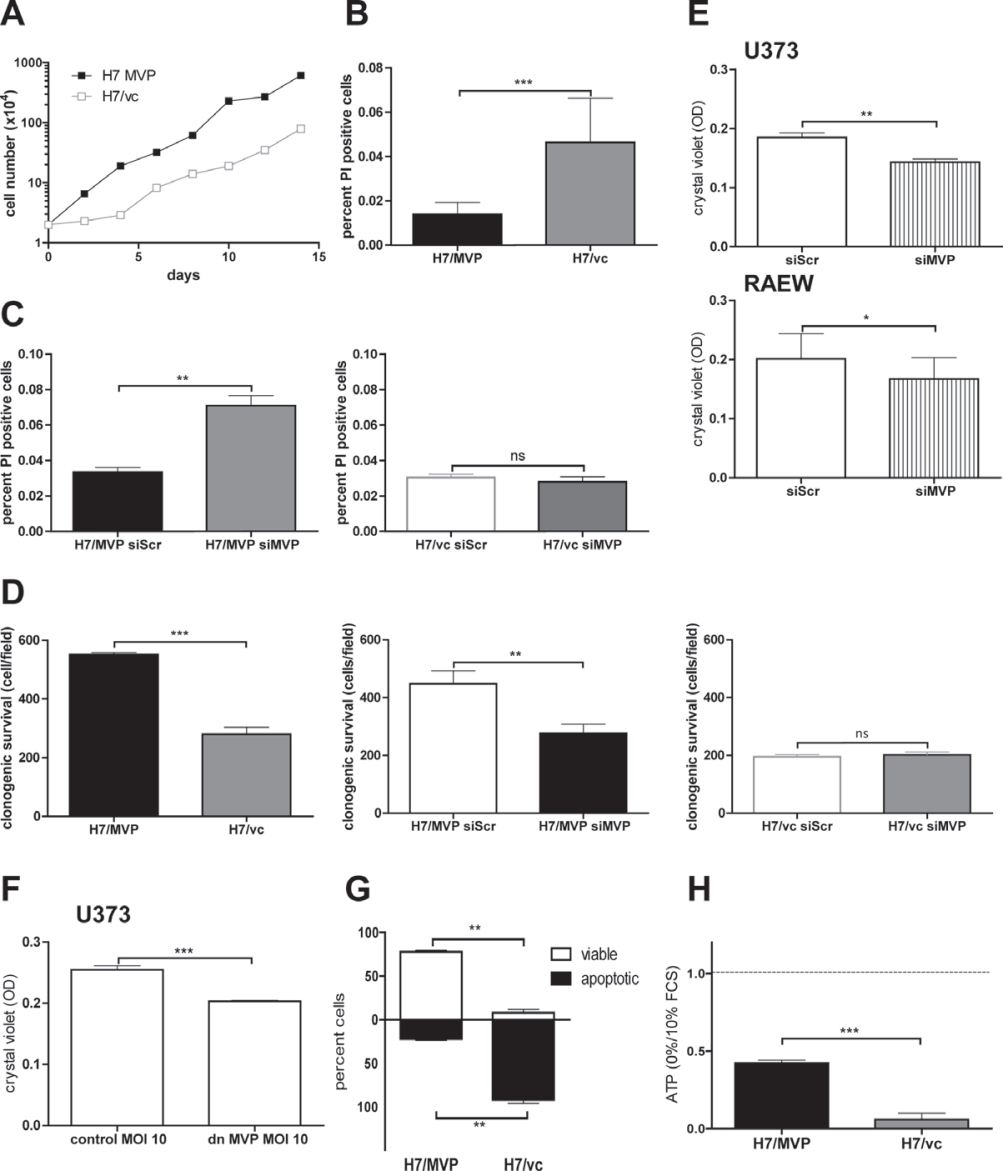
MVP supports GBM clonogenic survival and starvation resistance A. The growth pattern of H7/MVP and H7/vc cells seeded at low density (2×10^4^) in 6 well plates was monitored every other day for 2 weeks. The experiment was repeated twice leading to comparable results. B. and C. Spontaneous cell death was determined by Hoechst33258/propidium iodide staining at day three after seeding (B) or following siRNA-mediated MVP downregulation at day 4 after transfection (C). The percentage of PI-positive cells (dead) relative to the total cell number (Hoechst-positive) was calculated. D. Clonogenic survival potential was compared between H7/MVP and H7/vc cells 7 days after seeding (left panel) or 8 days after siRNA-mediated MVP down-modulation (middle and right panels). Bar graphs represent the cell number counted in 4 defined optical fields per well. E. Clonogenicity was determined in U373 and RAEW cells 8 days after siRNA-mediated MVP down-modulation. F. Impact of a dominant-negative MVP variant (dnMVP) on clonogenicity of U373 cells was determined 8 days after infection. E and F. The crystal violet absorbance was determined as optical density (OD) at 560nm. B-F. Bar graphs represent the means ± SD of three independent experiments (Student's t-test). G and H. Impact of long-term serum withdrawal (0% FCS; 5 days) on cell survival was analysed by Hoechst33258/propidium iodide staining (G) and ATP assay (H, unstarved controls are set as 1). Student's t-tests of data from three independent experiments in duplicates.

MVP overexpression did not alter cell proliferation rate at standard culture conditions in 10% foetal calf serum (FCS) (data not shown). However, after low density seeding, cell proliferation recovered more quickly in MVP-expressing cells (Figure 3A) corresponding to a reduced tendency towards spontaneous cell death induction (Figure 3B). Accordingly, only in the MVP-positive GBM cell models knockdown of MVP moderately but significantly upregulated the spontaneous cell death rate (Figure 3C; Supplementary Figure S2B). This suggests that vaults support GBM cell survival specifically under the stress of low density seeding. Correspondingly, MVP overexpression resulted in distinctly enhanced clonogenic survival of H7 cells which was significantly reversible by MVP siRNA (Figure 3D). In accordance, interference with endogenous MVP expression/function by siRNA or dnMVP specifically reduced clonogenic growth in all tested GBM cell lines (Figure 3E and F).

When the impact of MVP expression on GBM cell survival was tested under serum starvation stress, the differences became even more distinct. MVP-overexpressing H7 cells were mostly resistant against long-term starvation (75% viable cells after 5 days without serum and growth factors), whereas the majority (mean = 92%) of MVP-negative vector control cells underwent apoptosis in this setting (Figure 3G). Data from microscopic evaluation were confirmed by performing viability-indicative ATP assays (Figure 3H).

### MVP-mediated upregulation of the PI3K/AKT/mTOR pathway promotes migration and starvation resistance in human GBM cells

**Figure 4:**
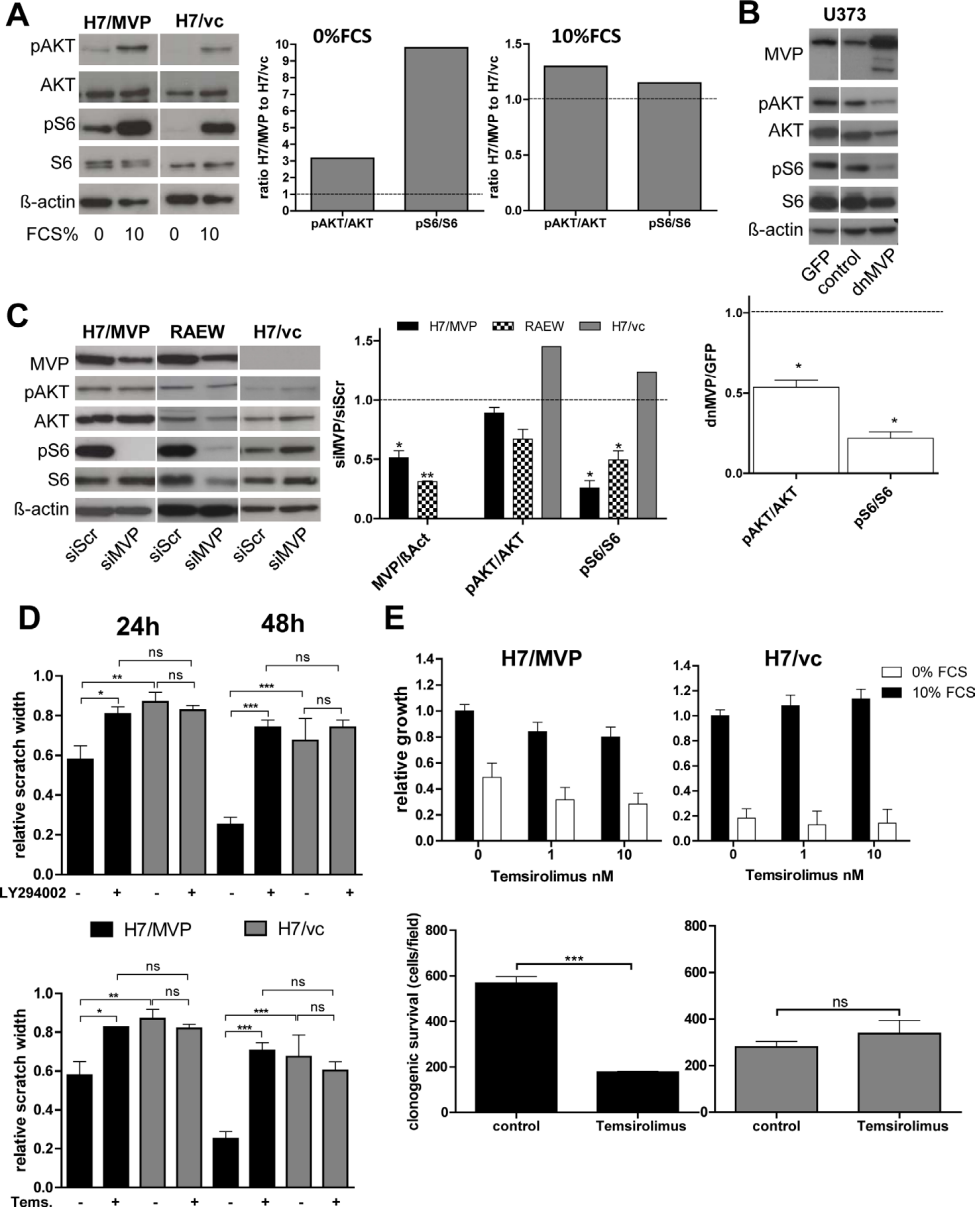
MVP supports migration and starvation resistance of GBM cells mainly via PI3K pathway upregulation A. Impact of serum withdrawal (72 hours) on the phosphorylation status of the indicated proteins was determined by Western blot analysis (left panel) and signals evaluated densitometrically (right panel). Data are given as ratios between phosphorylated and total proteins as indicated and values normalized to the respective H7/vc controls (dotted line). B. Impact of a dominant-negative MVP variant (dnMVP) on PI3K pathway activation was determined 72 hours after infection and compared to empty control or GFP-expressing viruses. Quantification (lower panel) was performed as under (A). C. Impact of siRNA-mediated MVP downregulation on MAPK and PI3K signalling under standard cell culture conditions was determined 96 hours after transfection. Quantification (right panel) was performed in each case relative to the respective siScr control. A-C. Statistical analyses were performed by one-sample t test. D. Effects of PI3K (2.5 μM LY294002) and mTOR (75 nM temsirolimus) inhibition on cell migration were determined at the indicated exposure times by wound healing assays (means ± SD of three experiments performed in duplicates; two-way ANOVA). E. Impacts of the mTOR inhibitor temsirolimus on cell viability at the indicated concentrations and FCS conditions (72 hours exposure time; upper panel) and on clonogenic survival (50 nM, 10% FCS, 6 days exposure; lower panel) were determined (means ± SD of three experiments; Student's t-test).

Vaults have been shown to interact with several intracellular signal cascades including the PI3K/AKT/mTOR pathway known to exert important roles in survival of diverse stress conditions and cell migration [20, 21]. Thus, we decided to investigate whether an impact on this pathway plays a role in MVP-mediated GBM cell survival and migration. Indeed, phosphorylation of the PI3K pathway mediators AKT and S6 was upregulated in total protein extracts of MVP-overexpressing H7 cells and this effect was more pronounced under serum withdrawal (Figure 4A). Correspondingly, blockade of MVP by dnMVP or siRNA (Figure 4B and C; respectively) reduced phosphorylation of AKT and even stronger of S6 in MVP-expressing cell models only. To analyse whether this PI3K pathway upregulation is involved in the MVP-mediated effects on GBM migration, scratch assays with the mTOR and PI3K inhibitors temsirolimus and LY294002, respectively, were performed. Both drugs completely reversed the higher migratory potential of H7/MVP cells to levels of the vector control (Figure 4D). Correspondingly, the profoundly enhanced survival of the MVP-expressing subline under serum withdrawal was almost completely reversed already by 1 nM temsirolimus (Figure 4E, upper panel). Similarly, clonogenic survival was significantly reduced by mTOR blockade specifically in the H7 MVP-positive background (Figure 4E, lower panel) and all endogenously MVP-expressing GBM cell models tested (RAEW representatively in Supplementary Figure S3).

### EGFR signals are involved in MVP-mediated stabilization of the PI3K/AKT/S6 signalling axis

**Figure 5:**
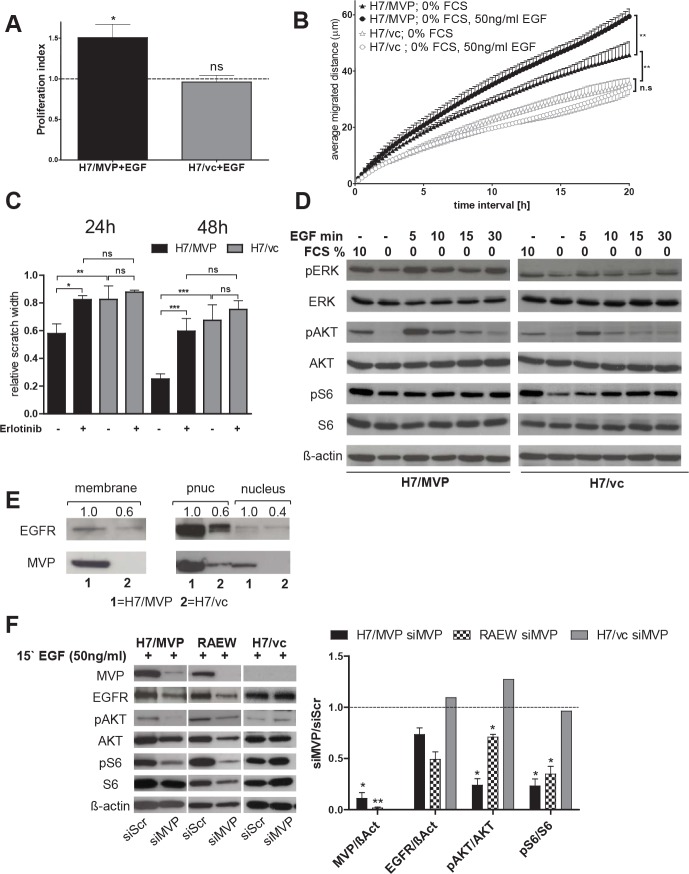
The EGF/EGFR signalling module is involved in MVP-mediated GBM cell survival, migration and PI3K pathway upregulation A. Impact of EGF stimulation on cell division under serum starvation was analysed as described in Material and Methods and values normalized to the respective controls. B. Distance of migration (in μm,) within 24 hours following EGF stimulation of serum-starved H7/MVP and H7/vc cells was determined by videomicroscopy. C. The effect of EGFR inhibition (2.5 μM erlotinib) on cell migration was determined by wound healing assays. B and C. Means ± SD of three experiments performed in duplicates; two-way ANOVA. D. Responsiveness of the MAPK and PI3K signal pathways to short-term EGF stimulation (50 ng/ml) of serum-starved cells. Quantification in Supplementary Figure S6A. E. EGFR and MVP contents of plasma membrane-enriched (membrane), nuclear (nucleus) and post-nuclear (pnuc) protein fractions as indicated. Purity controls compare Supplementary Figure S4. For quantification, band intensities were determined, ratios to ß-actin or lamin A/C calculated as appropriate and normalized to the respective values of H7/MVP cells. F. Exogenous and endogenous MVP in GBM cells (H7/MVP and RAEW, respectively) was knocked down by siRNA and 96 hours later the consequences on EGF responsiveness (15 minutes) analysed by immunoblot. Quantification of the indicated proteins (right panel) was performed in each case relative to the respective siScr controls. Statistical analyses were performed by one-sample t test.

As previous studies indicated binding and nuclear translocation of the negative PI3K pathway regulator phosphatase PTEN by MVP [22-24], we reasoned that this MVP function might underlie MVP-mediated PI3K pathway upregulation in human GBM cells. Indeed, PTEN levels of the MVP-overexpressing H7 subline were higher in the nuclear protein extracts but lower in 100000×g-precipitated membrane-enriched fraction as compared to vector control cells (Supplementary Figure S4). However when testing several inhibitors against different PI3K signalling cascade molecules, it turned out that MVP-overexpressing cells were not only hypersensitive against blockade of mTOR and PI3K, but also the EGFR inhibitor erlotinib (Supplementary Figure S5). This suggests that enhanced PI3K signalling in MVP-overexpressing cells might at least in part be based on hyperactivation of the EGFR. Accordingly, only the MVP-transfected H7 subline significantly responded to EGF stimulation by enhanced proliferation (Figure 5A). Additionally, the migratory phenotype of H7/MVP cells -detectable also under serum starvation-was markedly enhanced by EGF while H7/vc cells remained unresponsive (Figure 5B). Correspondingly, the EGFR inhibitor erlotinib, comparable to LY294002 and temsirolimus (compare Figure 4D), completely reversed the MVP-mediated migratory potential (Figure 5C). EGF stimulation of serum-starved cells induced hyperactivation of both the MAPK and PI3K pathways in the MVP-positive background (Figure 5D, quantification in Supplementary Figure S6A). Accordingly, we observed a MVP-mediated upregulation of EGFR in both membrane-enriched and postnuclear supernatant protein fractions of H7 cells (Figure 5E). Furthermore, video analysis of EGFP-MVP revealed enhanced translocation of vault particles to distinct membrane-subdomains and ruffling edges in response to EGF stimulation especially under starved conditions (Supplementary Figure S6B, Supplementary Movie 3 and 4). In contrast, siRNA-mediated downregulation of endogenous (RAEW) and transfected (H7/MVP) MVP under serum-starved, EGF-stimulated conditions resulted in decreased EGFR expression and reduced AKT and S6 phosphorylation (Figure 5F). Summarizing, these data suggest that MVP enhances responsiveness of human GBM cells to the EGF/EGFR signalling axis by upregulating EGFR levels at the GBM cell membrane.

### MVP overexpression in human GBM induces increased aggressiveness *in vivo*

**Figure 6:**
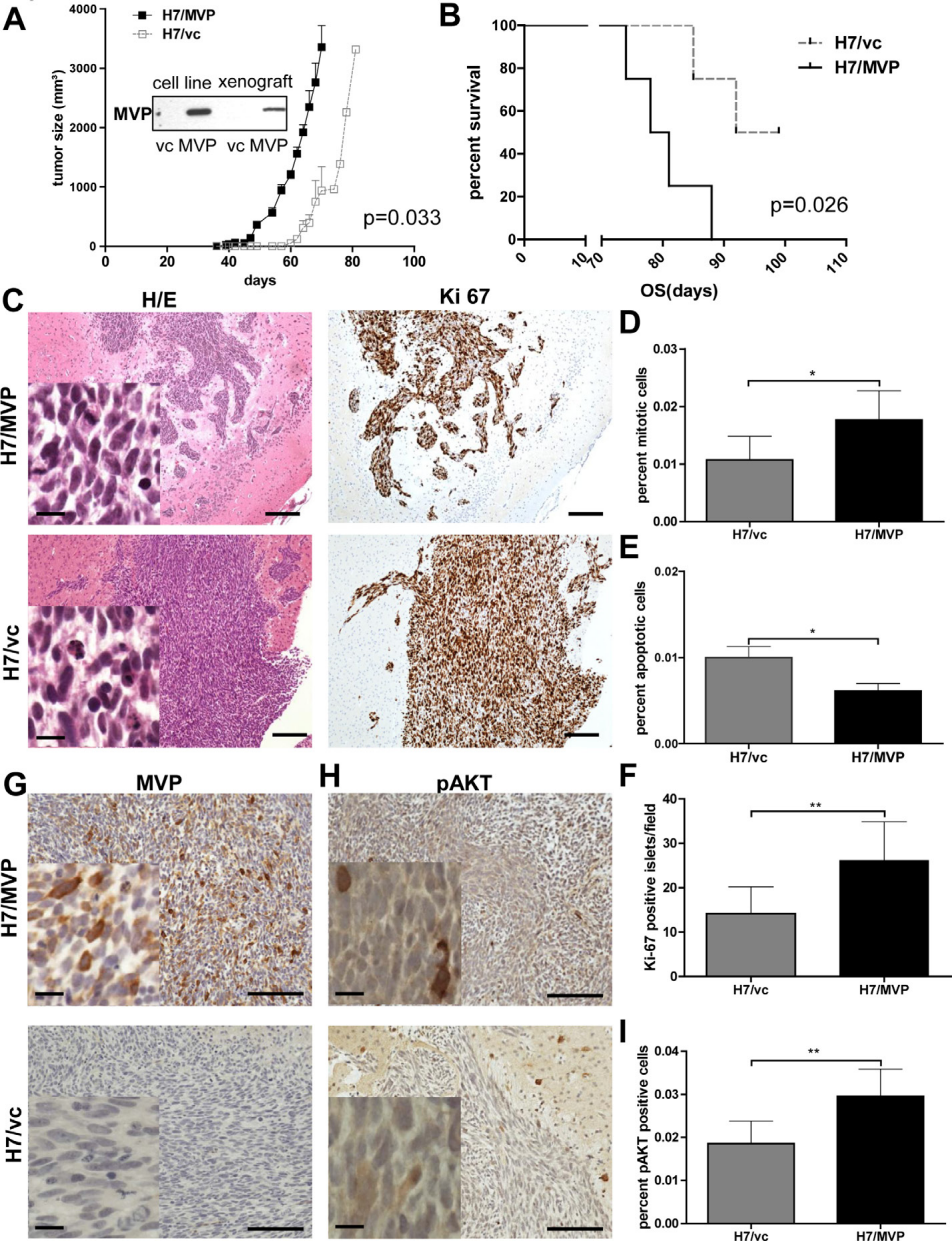
MVP supports GBM xenograft growth and invasiveness A and B. The impact of MVP status on subcutaneous tumour formation dynamics (A) and overall survival (OS) time of mice (B) after inoculation of 1 × 10^6^ H7/MVP and H7/vc cells is depicted. The Western blot in (A) demonstrates stable transgenic MVP expression during *in vivo* propagation. C – H. Impact of MVP on orthotopic GBM xenograft growth in the SCID mouse brain was tested by inoculation of 1 × 10^5^ and 1 × 10^6^ H7/MVP and H7/vc cells into the striatum. Tumours were obtained 10, 20 and 30 days post infection (dpi). C. Representative photomicrographs of H&E and Ki-67- (immuno)stained sections are shown (1 × 10^6^, 30dpi). In D and E the percentage of mitotic and apoptotic figures, respectively, were evaluated on H&E-stained tissue sections. F. Tumour invasiveness was quantified by evaluation of Ki-67-positive cancer cell islets without junction with the primary xenograft mass. G and H. Immunostainings for MVP (G) are opposed to immunohistochemical detection of phosphorylated AKT (pAKT) (H). Percentage of pAKT-positive cells is given in I. In case of (D), (E), (F) and (I) at least 6 sections derived from tumours 20 and 30 dpi (1 × 10^5^ and 1 × 10^6^ cells) were evaluated microscopically (means ± SD; Student's t-test). Scale bars are in C, G and H = 100 μm, 10 μm in the insets.

To test the *in vivo* relevance of the *in vitro* data, H7/MVP and vector control cells were transplanted both subcutaneously and orthotopically into SCID mice. At the subcutaneous location MVP induced significantly enhanced tumour growth of H7 cells and resulted in a distinctly reduced survival time of mice (Figure 6A and B). Protein extracts of tumours revealed stable overexpression of the MVP transgene during *in vivo* tumour formation (Western blot inset in Figure 6A). Accordingly, when injected orthotopically into the brain (Figure 6C) the number of mitotic cells (Figure 6D) was enhanced in histological sections of the MVP-positive cell model. Additionally, the number of apoptotic figures was, like under *in vitro* conditions, significantly reduced by MVP transfection (Figure 6E). Strikingly, MVP-overexpression induced distinct tissue invasion into normal brain with fissured margins and multiple Ki-67-positive tumour islets invading the surrounding brain parenchyma (Figure 6C and F). Immunohistochemical staining proved besides the stable expression of transfected MVP during xenograft growth (Figure 6G) also an enhanced number of cells stained positively for phosphorylated AKT (Figure 6H and 6I). Taken together these data suggest that MVP induces pro-survival and pro-migratory phenotypes in GBM cells leading to enhanced tumour aggressiveness *in vivo*.

## DISCUSSION

With regard to malignant progression, enhanced levels of MVP and vault particles have been reported in several cancer types and were occasionally associated with adverse prognosis [5]. Additionally, chemotherapy failure and radiation resistance have been frequently linked to enhanced MVP/vault expression [5, 25]. However a possible direct contribution of vaults to malignant phenotypes remains largely unexplored. Here we demonstrate that MVP supports aggressiveness of human GBM cells and xenografts by fostering an EGFR/PI3K/AKT pathway-mediated migratory/invasive potential as well as starvation resistance.

In a survey of human tissues and organs, brain was found to be characterized by comparably low MVP expression levels with only the microglia displaying clear-cut immunohistochemical staining [16, 26]. Thus, in an earlier study, we were surprised to detect an almost constitutive and intense overexpression of MVP in astrocytic brain tumour-derived cell lines [17]. In the present study, we now re-confirm activation of MVP gene expression in human gliomas as compared to normal brain based on extended expression array data sets. In accordance, overexpression of MVP in human glioma tissues has also been reported by other groups [18, 19]. The molecular mechanisms and signals underlying the activation of MVP expression in gliomas are still unknown. Interestingly, however, enhanced levels of MVP were repeatedly found in epileptic brain areas and considered as a possible factor causing failure of treatment with anti-epileptic drugs [27-29]. Moreover, considerable amounts of MVP were also present in explanted primary astrocytes suggesting that the induction of MVP expression might represent a more general stress-induced protection signal of neuroectodermal cells [17]. Accordingly, upregulation of MVP was also found as a consequence of neuronal/spinal injury in several model species including rat [30], electric ray [31] and zebrafish [32]. A direct functional involvement of MVP in neuroregeneration was proven in a spinal cord injury zebrafish model [32]. Together these observations implicate a stress/injury-inducible, survival- and migration-promoting function of vaults in the central nervous system.

With regard to gliomas, our present study suggests that such MVP-mediated migration- and survival-promoting functions are also activated during the malignant transformation/progression of astrocytes. Concerning cell migration, several previous studies reported that at different stages of evolution vaults are highly expressed in diverse cells with motile potential from *Dictyostelium* amoeba up to mature human dendritic cells, macrophages and the microglia [16, 26, 33, 34]. The hypothesis on a promigratory function of vaults, has now been proven in our GBM cell models by both up- and down-regulation of the migratory GBM cell potential following MVP overexpression and knock-down, respectively. Additionally, we observed distinctly enhanced brain invasion induced by MVP over-expression in an orthotopic glioblastoma xenograft. Consequently, GBM-associated MVP activation might have direct clinical relevance considering that diffuse invasion is, besides therapy resistance, the main cause of the devastating prognosis of GBM patients [35, 36].

A second distinct consequence of MVP overexpression was uncovered by culturing glioblastoma cells under serum-starved conditions. Both overexpression and knock-down experiments indicated a major role of MVP in preventing apoptosis induction as a consequence of growth factor deficiency. In accordance, MEFs from MVP-deficient mice were hypersensitive to serum withdrawal reacting with enhanced apoptosis induction [37]. Interestingly, also knock-out of the two MVP genes present in the slime mold *Dictyostelium discoideum* resulted in growth defects specifically under starvation conditions [33]. Together these observations suggest an evolutionary conserved role of vaults in starvation resistance. Additionally, Rhy et al. reported on enhanced MVP expression as a contributor to aging-associated apoptosis resistance against diverse stress signals in human fibroblasts [38]. These protective functions might be hijacked in the malignant tissue where cancer cells are constantly threatened by shortage in oxygen and nutrition.

One important intracellular signalling pathway known to be able to mediate both migratory competence and starvation resistance is the PI3K/mTOR/AKT cascade [20, 21] activated by diverse potentially oncogenic receptor tyrosine kinases (RTK) including the EGFR. With regard to GBM, inhibition of the PI3K pathway by knock-down strategies resulted in decreased proliferation, migration, and invasion [39]. Several previous observations have suggested that vaults might interfere with the PI3K cell survival signal axis. Thus an MVP-mediated, cyto-protective effect against hyperosmotic stress was found to be dependent on enhanced PI3K pathway activation in SW620 colon cancer cells [40]. Additionally, the first intracellular signalling regulator found to interact with MVP was the PI3K antagonist and tumour suppressor PTEN [24], frequently deregulated in human GBM by gene deletion, mutation, promoter methylation or miRNA mediated suppression [41, 42]. Subsequently, two studies by the group of C. Eng reported MVP-mediated nuclear translocation of PTEN which might sequester this phosphatase away from its lipid substrates at the cell membrane [22, 23]. In accordance with these data, we found enhanced levels of PTEN in nuclear extracts but reduced levels in membrane-enriched fractions of MVP-transfected GBM cells. These data suggest that vaults might support PI3K activation by PTEN inhibition. However, small molecule kinase inhibitor studies revealed that not only blockade of PI3K and mTOR but also of EGFR, one of the major oncogenic RTK molecules in human GBM [43], completely reversed the effects of MVP on GBM cell migration and starvation resistance. Thus we hypothesized that EGFR hyperactivation might synergize with PTEN inhibition in MVP-mediated PI3K signal stabilization and consequently GBM aggressiveness. Accordingly, regulation of MVP tyrosyl-phosphorylation by SHP-2 was suggested to support EGF-mediated MAPK pathway activation but probably also IGF-driven PI3K/AKT survival signals in fibroblasts [37]. Indeed, we found that MVP has a profound impact on EGFR expression levels and EGF-responsiveness of our GBM cell models. Interestingly, this seems to be a lineage-specific effect since in the case of hepatoma cell lines we observed gefitinib resistance as a consequence of MVP overexpression [44]. This might rely on the fact that alterations in EGFR are pivotal and very frequent in primary human GBM but not hepatoma. Accordingly, an association between MVP expression and the proteomic signalling network activated by the oncogenic EGFRvIII variant in GBM cells was reported recently [19]. Video analysis of GBM cells revealed that the transport of vault particles to distinct membrane-subdomains and ruffling edges was enhanced in response to EGF stimulation especially under growth factor-starved conditions. This suggests that MVP might directly interact with EGFR or a binding partner leading to enhanced protein stabilization or altered receptor recycling rates. These possibilities are addressed by on-going projects in our lab.

Taken together our data demonstrate, to the best of our knowledge for the first time, that vault particles contribute to cancer aggressiveness. We found that MVP expression is almost constitutively activated during malignant transformation in tumours of astrocytic origin and supports EGFR/PI3K signalling pathway activation leading to enhanced starvation resistance and migration/invasion potential. A possible quality of MVP overexpression as predictive marker for GBM responsiveness to EGFR, PI3K or mTOR targeting agents, as suggested by our transgenic cell models, needs to be addressed using well-characterized patient material from controlled clinical studies.

## MATERIALS AND METHODS

### Drugs and chemicals

Temsirolimus was obtained from Wyeth Pharmaceuticals Inc. Erlotinib was generously supplied by Roche and LY294002 was obtained from LC Labs. All solutions were freshly prepared before use. All other drugs were purchased from Sigma.

### Cell lines, primary cell cultures and human tissue

U373 GBM and A549 NSCLC cells were obtained from the ATCC and grown in minimal essential medium (MEM) (PAA) containing 10% FCS (PAA), 1 mM sodium pyruvate and 1% non-essential amino acids and in RPMI-1640 medium (PAA) with 10% FCS, respectively. RAEW cells, established at the Department of Neurosurgery, Wagner-Jauregg Hospital in Linz, were cultured in RPMI-1640. Furthermore, MR-1 GBM cells were kindly provided by Dr. T. Kurata, Tokyo, Japan and were grown in RPMI-1640 with 10% FCS. The H7 GBM cell line was established at the Institute of Cancer Research, Vienna from a 39 years old male patient diagnosed with GBM. All cell lines and subclones used were authenticated by array-comparative genomic hybridization [45] and DNA fingerprinting and periodically checked for *Mycoplasma* contamination (Mycoplasma Stain Kit; Sigma).

### Stable MVP expression

Stable MVP-overexpressing H7 cell clones and the corresponding vector control cells were established as described previously [46].

### Silencing of MVP

For transient gene silencing cells were transduced with ON-TARGETplus SmartPOOL siRNA for MVP or for control siRNA (Dharmacon) as described [44]. Oligofectamine (Life Technologies) was used as transfection reagent.

### Migration assays

Cell migration was tested by wound-healing or transwell-chamber assays. Confluent monolayers were wounded using a sterile 200 μl tip and treated with the respective kinase inhibitors (erlotinib, LY294002 and temsirolimus). Photomicrographs from at least three fixed positions of scratches were taken at 0, 24 and 48 hours on a Nikon Eclipse inverted microscope. Gap-width was determined using Tscratch software [47]. Results were calculated as percentage open area relative to the starting gaps after 24 and 48 hours. Transwell-chamber assays were performed as published [48]. The migratory potential was analysed by counting crystal violet-positive cells of at least 4 defined fields at the bottom of each filter (Image J software, National Institutes of Health). Alternatively, migrated cells were destained using a 2% SDS solution and absorbance of free crystal violet was measured at 560nm. All experiments were performed in duplicates and repeated at least twice.

### Videomicroscopy analysis

For migration analysis [49] 50 000 cells were plated in each well of a 24-well plate with complete growth medium. After overnight cell attachment the medium was changed to CO_2_-independent medium (Gibco-BRL Life Technologies, UK) without FCS but supplemented with 4mM glutamine. The culture plate was kept in a 37 °C custom designed incubator built around an inverted phase-contrast microscope (World Precision Instruments). Images of 3 neighbouring microscopic fields were taken at five-minute intervals for 1 day before and 2 days after treatment with 50ng/ml EGF. Images from the phase contrast microscope were analysed for migration data with a cell-tracking program enabling manual marking of individual cells and recording their position parameters into data files. The parameter “migrated distance” was calculated by averaging for each cell the displacement for each time interval up to 24 hours. For each condition at least 20 cells were tracked from different fields of view and all experiments were repeated twice. Cell proliferation index was also assessed on the basis of the videos by counting cell divisions within the first 48 hours after EGF stimulation.

### Protein isolation, cell fractionation and Western blot analysis

Total cellular protein extracts were isolated from untreated semiconfluent cell cultures grown in T-25 cm^2^ flasks. For knock-down experiments proteins were extracted 4 days after siRNA transfection or adenoviral infection. After evaluation of protein concentrations using the Micro BCA protein assay reagent kit (Pierce), immunoblots were performed as described [44]. Isolation of 100000×g protein fractions and sucrose gradient centrifugation were performed as described [10, 50]. Cell membranes were isolated as published [51]. For extraction of postnuclear supernatant and nuclear protein fractions the NE-PER Nuclear and Cytoplasmic Extraction kit was used (Thermo Fisher Scientific). The following primary antibodies were diluted 1 : 1000 in TBS with 3% BSA and 0,1% Tween: MVP monoclonal mouse clone 42 (Transduction Laboratories), phospho-ERK1/2 (Thr202/Tyr204), ERK1/2, phospho-S6 ribosomal protein (Ser240/244), S6, phospho-AKT (Ser473), AKT, PTEN and EGFR were all purchased from Cell Signaling Technology. ß-actin AC-15 and lamin A/C antibodies were both obtained from Sigma. The immunoreactive bands were detected with horseradish peroxidase-labelled antibodies from Santa Cruz Biotechnology. For band quantification the QuantiScan software (Biosoft) was used.

### Immunohistochemistry and immunofluorescence

Consecutive tissue sections from formalin-fixed and paraffin-embedded brain specimens were deparaffinised and rehydrated. After heating for 10 min in 10mM citrate buffer (pH 6.0), the slides were incubated overnight at 4°C with LRP-56 (for MVP, 1:20; Santa Cruz Biotechnology) and p-AKT (Ser473; 1:25; Cell Signalling Technology) antibodies, respectively. To evaluate proliferation, sections were stained with the Ki-67 (clone MiB-1) antibody from DAKO (Glostrup, Denmark; 1: 100) for 1 hour at room temperature. Binding of primary antibodies was detected with the UltraVision LP detection system according to the manufacturer's instructions (Thermo Fisher Scientific), followed by incubation with 3,3′-diaminobenzidine and counterstaining with haematoxylin. Haematoxylin and eosin (H&E) staining were used to localize the tumour mass in the brain and to count the number of apoptotic and mitotic cells. The percentage of pAKT-positive, apoptotic and mitotic cells were evaluated by counting at least four visual fields per slide microscopically. With regard to invasive potential, the number of Ki-67-positive islets without junction with the primary tumour mass of at least 6 slides from 2 time points (20 and 30 days after orthotopic transplantation) was determined.

For immunofluorescence cells were grown in chamber slides and after attachment fixed with 4% formalin/phosphate-buffered saline (PBS) followed by permeabilization with 0.5% TritonX/PBS. All further procedures were done as described [15]. The following primary and secondary antibodies were used: LRP-56 and PTEN as listed above, (all 1:50 in PBS/1% BSA), fluorescein isothiocyanate (FITC)-labelled anti-mouse (F-1010) or anti-rabbit (F-9887) and tetramethyl rhodamine isothiocyanate (TRITC)-labelled anti-mouse (T6528) (all 1:500; Sigma). Actin filaments were visualized with FITC-phalloidin staining (1:500; Sigma). Stained cells were mounted with DAPI (Vectorshield) and further analysed using a confocal laser scanning microscope (Zeiss Invert Axio Observer.Z1, Two-channel LSM 700 URGB) equipped with LD LCI Plan-Apochromat 25×/0.8 Imm Korr DIC M27 and EC Plan-Neofluar 40×/1.3 Oil DIC M27 objective lenses. Digital images were taken using the Zeiss ZEN (2011) software.

### Cell viability, cell death, and clonogenic assays

For cell viability 3 × 10^3^ cells were seeded into 96-well plates and 24 hours later cells were exposed to drugs as indicated. Activity of anticancer drugs was screened by MTT assay (EZ4U, Biomedica). Additionally, cell survival was measured 5 days after serum withdrawal by CellTiter-Glo® Luminescent Cell Viability assay (Promega). Cell death rates were determined by simultaneous staining with Hoechst33258 and propidium iodide as published [52]. For clonogenic assays, cells were seeded (in the respective experiments 24 hours after transfection or viral infection) at a density of 3 × 10^3^ cells per well into 6-well plates and monitored for at least 7 days. In drug exposure experiments, 24 hours after seeding cells were treated with the respective drugs for 6 days. Cells were stained with crystal violet and either counted using Image J software, or quantified by densitometry as described above.

### Subcutaneous and orthotopic xenografts

Six- to 8-week-old female, immunodeficient C.B-17/IcrHsd-Prkcdscid SCID mice were purchased from Harlan and kept in a pathogen-free environment. For subcutaneous tumour formation 1 × 10^6^ H7/MVP and H7/vc cells were injected into the right flanks of mice. Animals were continually controlled for distress development and tumour size was determined as described [48]. Orthotopically growing brain tumours were generated by injection of 1 × 10^5^ or 1 × 10^6^ H7/MVP and H7/vc cells into the striatum of mouse brain as described earlier [53]. Cell injection was carried out in anaesthesized animals using Lab Standard Stereotaxic Instrument supplied with Quintessential Stereotaxic Injector (Stoelting) at constant speed 0.5 μl/min. At 10, 20 and 30 days after cell injection mice were euthanized under anaesthesia by transcardial perfusion using PBS and Accustain solution (Sigma). Brains were post-fixed by immersion in the Accustain fixative for at least 12 hours at 4 °C. After paraffin embedding, tissues were further analysed. All experiments were performed according to the Federation of Laboratory Animal Science Association guidelines for the use of experimental animals and approved by the institutional ethics committee and Austrian government authorities (BMWF-68.205/0064-II/3b/2011 and BMWF-66.009/0157-II/10b/2008).

### Statistical analysis

All data are expressed as mean ± S.D. Statistical significance of differences was analysed by using unpaired Student's t-test or two-way ANOVA, as appropriate followed by Bonferroni post-tests. Survival analysis was performed using Kaplan–Meier curves. A *p*-value < 0.05 was considered statistically significant. Throughout the study the following classification is used: *, *p* < 0.05; **, *p* < 0.01 ***, *p* < 0.001.

### Other methods

Information of other methods performed, including cloning strategies for MVP, EGFP-MVP and dnMVP, quantitative reverse transcription-polymerase chain reaction and gene expression analysis are provided as Supplementary Material and Methods.

## Supplementary Figures, Methods and Movies













## References

[1] Stupp R, Hegi ME, Mason WP, van den Bent MJ, Taphoorn MJ, Janzer RC, Ludwin SK, Allgeier A, Fisher B, Belanger K, Hau P, Brandes AA, Gijtenbeek J, Marosi C, Vecht CJ, Mokhtari K (2009). Effects of radiotherapy with concomitant and adjuvant temozolomide versus radiotherapy alone on survival in glioblastoma in a randomised phase III study: 5-year analysis of the EORTC-NCIC trial. Lancet Oncol.

[2] Wen PY, Kesari S (2008). Malignant gliomas in adults. N Engl J Med.

[3] Teodorczyk M, Martin-Villalba A (2010). Sensing invasion: cell surface receptors driving spreading of glioblastoma. J Cell Physiol.

[4] Bredel M (2001). Anticancer drug resistance in primary human brain tumors. Brain Res Brain Res Rev.

[5] Steiner E, Holzmann K, Elbling L, Micksche M, Berger W (2006). Cellular functions of vaults and their involvement in multidrug resistance. Curr Drug Targets.

[6] Rome L, Kedersha N, Chugani D (1991). Unlocking vaults: organelles in search of a function. Trends Cell Biol.

[7] Kickhoefer VA, Vasu SK, Rome LH (1996). Vaults are the answer, what is the question?. Trends Cell Biol.

[8] van Zon A, Mossink MH, Scheper RJ, Sonneveld P, Wiemer EA (2003). The vault complex. Cell Mol Life Sci.

[9] Berger W, Steiner E, Grusch M, Elbling L, Micksche M (2009). Vaults and the major vault protein: novel roles in signal pathway regulation and immunity. Cell Mol Life Sci.

[10] Kickhoefer VA, Rajavel KS, Scheffer GL, Dalton WS, Scheper RJ, Rome LH (1998). Vaults are up-regulated in multidrug-resistant cancer cell lines. J Biol Chem.

[11] Mossink MH, van Zon Zon, Scheper RJ, Sonneveld P, Wiemer EA, Schoester M, Houtsmuller AB, Scheffer GL, Franzel-Luiten E, Kickhoefer VA, Mossink M, Poderycki MJ, Chan EK, Rome LH (2003). Vaults: a ribonucleoprotein particle involved in drug resistance?. Oncogene.

[12] Park K (2012). The role of major vault protein (MVP) in drug resistance. J Control Release.

[13] Suprenant KA (2002). Vault ribonucleoprotein particles: sarcophagi, gondolas, or safety deposit boxes?. Biochemistry.

[14] Rome LH, Kickhoefer VA (2012). Development of the Vault Particle as a Platform Technology. ACS Nano.

[15] Steiner E, Holzmann K, Pirker C, Elbling L, Micksche M, Sutterluty H, Berger W (2006). The major vault protein is responsive to and interferes with interferon-gamma-mediated STAT1 signals. J Cell Sci.

[16] Izquierdo MA, Scheffer GL, Flens MJ, Giaccone G, Broxterman HJ, Meijer CJ, van der Valk P, Scheper RJ (1996). Broad distribution of the multidrug resistance-related vault lung resistance protein in normal human tissues and tumors. Am J Pathol.

[17] Berger W, Spiegl-Kreinecker S, Buchroithner J, Elbling L, Pirker C, Fischer J, Micksche M (2001). Overexpression of the human major vault protein in astrocytic brain tumor cells. Int J Cancer.

[18] Zhang R, Tremblay TL, McDermid A, Thibault P, Stanimirovic D (2003). Identification of differentially expressed proteins in human glioblastoma cell lines and tumors. Glia.

[19] Johnson H, Del Rosario Rosario, Bryson BD, Schroeder MA, Sarkaria JN, White FM (2012). Molecular characterization of EGFR and EGFRvIII signaling networks in human glioblastoma tumor xenografts. Mol Cell Proteomics.

[20] Xue G, Hemmings BA (2013). PKB/Akt-Dependent Regulation of Cell Motility. J Natl Cancer Inst.

[21] Zoncu R, Efeyan A, Sabatini DM (2011). mTOR: from growth signal integration to cancer, diabetes and ageing. Nat Rev Mol Cell Biol.

[22] Chung JH, Ginn-Pease ME, Eng C (2005). Phosphatase and tensin homologue deleted on chromosome 10 (PTEN) has nuclear localization signal-like sequences for nuclear import mediated by major vault protein. Cancer Res.

[23] Minaguchi T, Waite KA, Eng C (2006). Nuclear localization of PTEN is regulated by Ca(2+) through a tyrosil phosphorylation-independent conformational modification in major vault protein. Cancer Res.

[24] Yu Z, Fotouhi-Ardakani N, Wu L, Maoui M, Wang S, Banville D, Shen SH (2002). PTEN associates with the vault particles in HeLa cells. J Biol Chem.

[25] Lara PC, Pruschy M, Zimmermann M, Henriquez-Hernandez LA (2011). MVP and vaults: a role in the radiation response. Radiat Oncol.

[26] Chugani DC, Kedersha NL, Rome LH (1991). Vault immunofluorescence in the brain: new insights regarding the origin of microglia. J Neurosci.

[27] Aronica E, Gorter JA, Ramkema M, Redeker S, Ozbas-Gercerer F, van Vliet EA, Scheffer GL, Scheper RJ, van der Valk P, Baayen JC, Troost D (2004). Expression and cellular distribution of multidrug resistance-related proteins in the hippocampus of patients with mesial temporal lobe epilepsy. Epilepsia.

[28] Liu B, Wang T, Wang L, Wang C, Zhang H, Gao GD (2011). Up-regulation of major vault protein in the frontal cortex of patients with intractable frontal lobe epilepsy. J Neurol Sci.

[29] van Vliet EA, Aronica E, Redeker S, Gorter JA (2004). Expression and cellular distribution of major vault protein: a putative marker for pharmacoresistance in a rat model for temporal lobe epilepsy. Epilepsia.

[30] Komori N, Takemori N, Kim HK, Singh A, Hwang SH, Foreman RD, Chung K, Chung JM, Matsumoto H (2007). Proteomics study of neuropathic and nonneuropathic dorsal root ganglia: altered protein regulation following segmental spinal nerve ligation injury. Physiol Genomics.

[31] Li JY, Volknandt W, Dahlstrom A, Herrmann C, Blasi J, Das B, Zimmermann H (1999). Axonal transport of ribonucleoprotein particles (vaults). Neuroscience.

[32] Pan HC, Lin JF, Ma LP, Shen YQ, Schachner M (2012). Major vault protein promotes locomotor recovery and regeneration after spinal cord injury in adult zebrafish. Eur J Neurosci.

[33] Vasu SK, Rome LH (1995). Dictyostelium vaults: disruption of the major proteins reveals growth and morphological defects and uncovers a new associated protein. J Biol Chem.

[34] Mossink MH, De Groot Groot, Van Zon Zon, Franzel-Luiten E, Schoester M, Scheffer GL, Sonneveld P, Scheper RJ, Wiemer EA (2003). Unimpaired dendritic cell functions in MVP/LRP knockout mice. Immunology.

[35] Huse JT, Holland E, Deangelis LM (2012). Glioblastoma: Molecular Analysis and Clinical Implications. Annu Rev Med.

[36] Furnari FB, Fenton T, Bachoo RM, Mukasa A, Stommel JM, Stegh A, Hahn WC, Ligon KL, Louis DN, Brennan C, Chin L, DePinho RA, Cavenee WK (2007). Malignant astrocytic glioma: genetics, biology, and paths to treatment. Genes Dev.

[37] Kolli S, Zito CI, Mossink MH, Wiemer EA, Bennett AM (2004). The major vault protein is a novel substrate for the tyrosine phosphatase SHP-2 and scaffold protein in epidermal growth factor signaling. J Biol Chem.

[38] Ryu SJ, An HJ, Oh YS, Choi HR, Ha MK, Park SC (2008). On the role of major vault protein in the resistance of senescent human diploid fibroblasts to apoptosis. Cell Death Differ.

[39] Weber GL, Parat MO, Binder ZA, Gallia GL, Riggins GJ (2011). Abrogation of PIK3CA or PIK3R1 reduces proliferation, migration, and invasion in glioblastoma multiforme cells. Oncotarget.

[40] Ikeda R, Iwashita K, Sumizawa T, Beppu S, Tabata S, Tajitsu Y, Shimamoto Y, Yoshida K, Furukawa T, Che XF, Yamaguchi T, Ushiyama M, Miyawaki A, Takeda Y, Yamamoto M, Zhao HY (2008). Hyperosmotic stress up-regulates the expression of major vault protein in SW620 human colon cancer cells. Exp Cell Res.

[41] Koul D (2008). PTEN signaling pathways in glioblastoma. Cancer Biol Ther.

[42] Li H, Yang BB (2012). Stress response of glioblastoma cells mediated by miR-17-5p targeting PTEN and the passenger strand miR-17-3p targeting MDM2. Oncotarget.

[43] Taylor TE, Furnari FB, Cavenee WK (2012). Targeting EGFR for treatment of glioblastoma: molecular basis to overcome resistance. Curr Cancer Drug Targets.

[44] Losert A, Lotsch D, Lackner A, Koppensteiner H, Peter-Vorosmarty B, Steiner E, Holzmann K, Grunt T, Schmid K, Marian B, Grasl-Kraupp B, Schulte-Hermann R, Krupitza G, Berger W, Grusch M (2012). The major vault protein mediates resistance to epidermal growth factor receptor inhibition in human hepatoma cells. Cancer Lett.

[45] Mathieu V, Pirker C, Schmidt WM, Spiegl-Kreinecker S, Lotsch D, Heffeter P, Hegedus B, Grusch M, Kiss R, Berger W (2012). Aggressiveness of human melanoma xenograft models is promoted by aneuploidy-driven gene expression deregulation. Oncotarget.

[46] Holzmann K, Ambrosch I, Elbling L, Micksche M, Berger W (2001). A small upstream open reading frame causes inhibition of human major vault protein expression from a ubiquitous mRNA splice variant. FEBS Lett.

[47] Geback T, Schulz MM, Koumoutsakos P, Detmar M (2009). TScratch: a novel and simple software tool for automated analysis of monolayer wound healing assays. Biotechniques.

[48] Fischer H, Taylor N, Allerstorfer S, Grusch M, Sonvilla G, Holzmann K, Setinek U, Elbling L, Cantonati H, Grasl-Kraupp B, Gauglhofer C, Marian B, Micksche M, Berger W (2008). Fibroblast growth factor receptor-mediated signals contribute to the malignant phenotype of non-small cell lung cancer cells: therapeutic implications and synergism with epidermal growth factor receptor inhibition. Mol Cancer Ther.

[49] Hegedus B, Zach J, Czirok A, Lovey J, Vicsek T (2004). Irradiation and Taxol treatment result in non-monotonous, dose-dependent changes in the motility of glioblastoma cells. J Neurooncol.

[50] Berger W, Elbling L, Micksche M (2000). Expression of the major vault protein LRP in human non-small-cell lung cancer cells: activation by short-term exposure to antineoplastic drugs. Int J Cancer.

[51] Heinzle C, Gsur A, Hunjadi M, Erdem Z, Gauglhofer C, Stattner S, Karner J, Klimpfinger M, Wrba F, Reti A, Hegedus B, Baierl A, Grasl-Kraupp B, Holzmann K, Grusch M, Berger W (2012). Differential Effects of Polymorphic Alleles of FGF Receptor 4 on Colon Cancer Growth and Metastasis. Cancer Res.

[52] Kowol CR, Heffeter P, Miklos W, Gille L, Trondl R, Cappellacci L, Berger W, Keppler BK (2012). Mechanisms underlying reductant-induced reactive oxygen species formation by anticancer copper(II) compounds. J Biol Inorg Chem.

[53] Hlavaty J, Jandl G, Liszt M, Petznek H, Konig-Schuster M, Sedlak J, Egerbacher M, Weissenberger J, Salmons B, Gunzburg WH, Renner M (2011). Comparative evaluation of preclinical in vivo models for the assessment of replicating retroviral vectors for the treatment of glioblastoma. J Neurooncol.

