# Supplementary material for: Major vault protein supports glioblastoma survival and migration by upregulating the EGFR/PI3K signalling axis
Source: Oncotarget. 2013 Sep 1;4(11):1904–18. doi: 10.18632/oncotarget.1264 (PMC3875758; doi:10.18632/oncotarget.1264)
Supplement: Supplementary file 1 [file oncotarget-04-1904-s001.pdf]

Major vault protein supports glioblastoma survival and migration by upregulating the EGFR/PI3K signalling axis - Lötsch et al

A

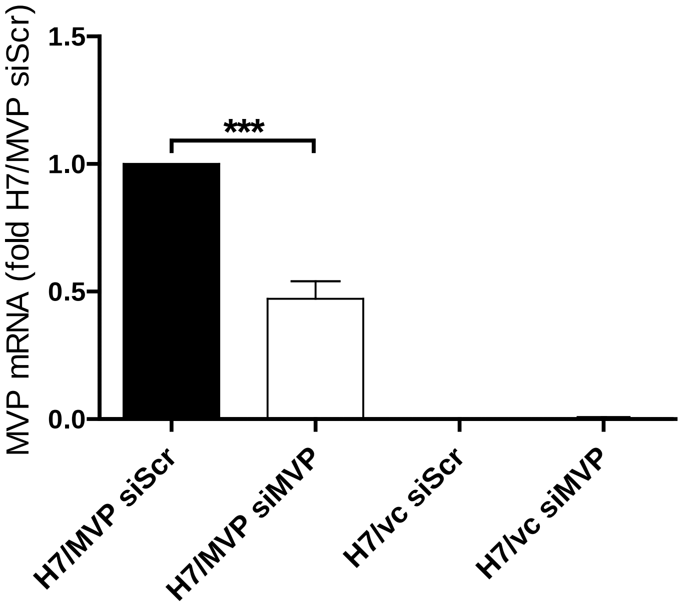

B

H7/vc

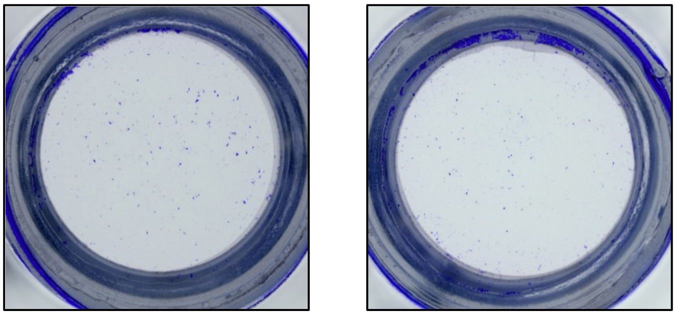

Figure S1: **MVP expression manipulation by siRNA and impact on cell migration.** A. The extent of MVP mRNA reduction induced by the indicated siRNA 48 hours after transfection was analysed by real-time PCR and compared to scramble siRNA (siScr) set as 1. Statistical analysis was performed by one-way ANOVA and Bonferroni post-test. B. Photomicrographs reflecting the incapacity of H7/vc cells migrated through the pores of transwell-filters 96 hours after siRNA transfection. Due to lack of migrated cells quantification was impossible.

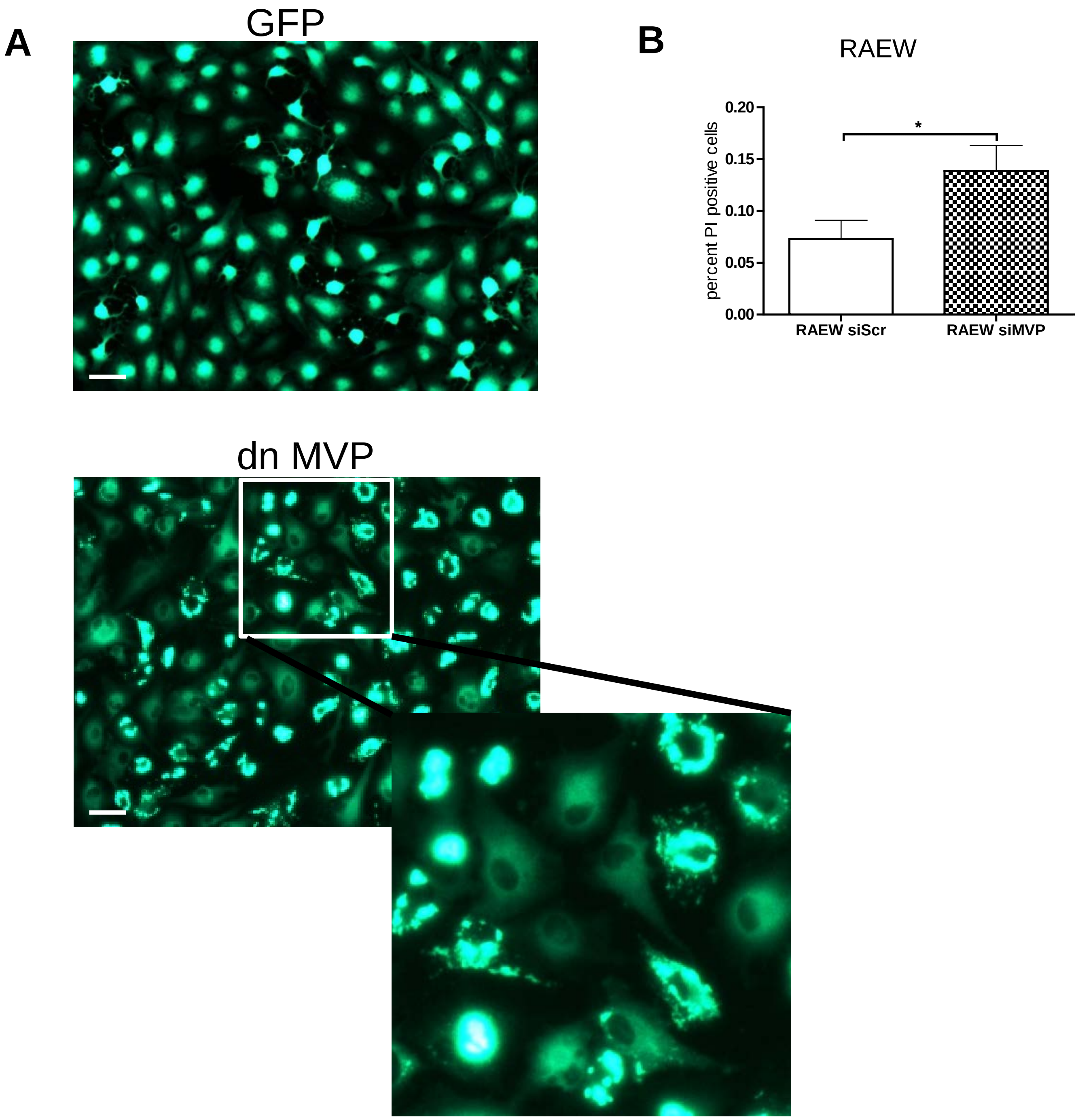

Figure S2: **Blockade of endogenous MVP and cell death induction.** A. Photomicrographs of U373 cells were taken 24 hours after infection with an adenovirus encoding a GFP-tagged dominant-negative MVP variant (upper panel) or GFP alone (lower panel) (scale bar = 10 μm). B. Impact of MVP-directed siRNA on the cell death rate of RAEW cells, expressing high level of endogenous MVP, was determined by Hoechst33258/propidium iodide (PI) staining at day 4 after transfection. The percentage of PI-positive cells (dead) relative to the total cell number (Hoechst33258-positive) was calculated.

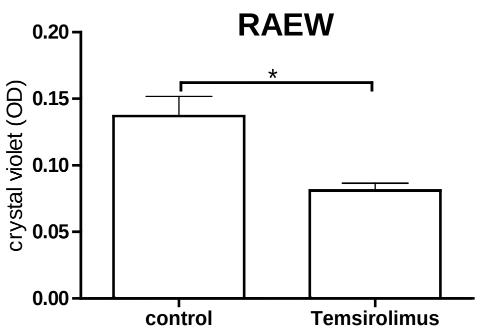

Figure S3: **MVP expression and the PI3K pathway in GBM.** Impact of mTOR inhibition with 50 nM temsirolimus on clonogenic survival of RAEW cells within 6 days (means ± SD of three experiments; Student´s t-test) was determined.

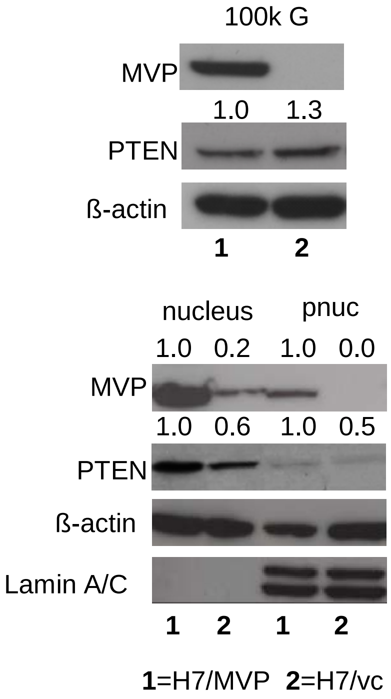

Figure S4: MVP overexpression and the impact on PTEN distribution. The subcellular localization of PTEN was determined by Western blot. Membrane/particulate fraction isolated after 100000xg centrifugation (100 kG) as well as postnuclear supernatant (pnuc) and nuclear protein fractions were analysed. Lamin A/C served as purity control marker for nuclear protein extracts. For quantification, respective band intensities were determined, ratios to ß-actin or lamin A/C calculated as appropriate and normalized to the respective values of H7/MVP cells.

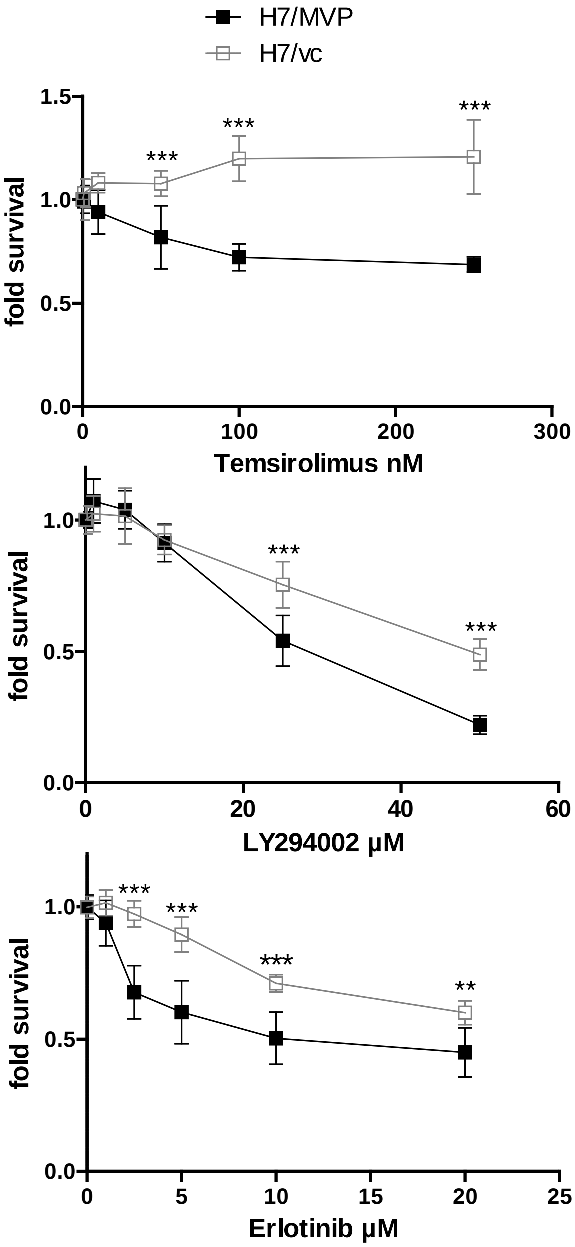

Figure S5: Impact of MVP on GBM cell responsiveness against kinase inhibitors. Dose-response curves of H7/MVP and H7/vc cells were established for the mTOR inhibitor temsirolimus, the PI3K inhibitor LY294002 and the EGFR inhibitor erlotinib. Following 72 hours drug exposure cell viability was determined by MTT assays. Statistical analyses were performed by two-way ANOVA and Bonferroni post-test.

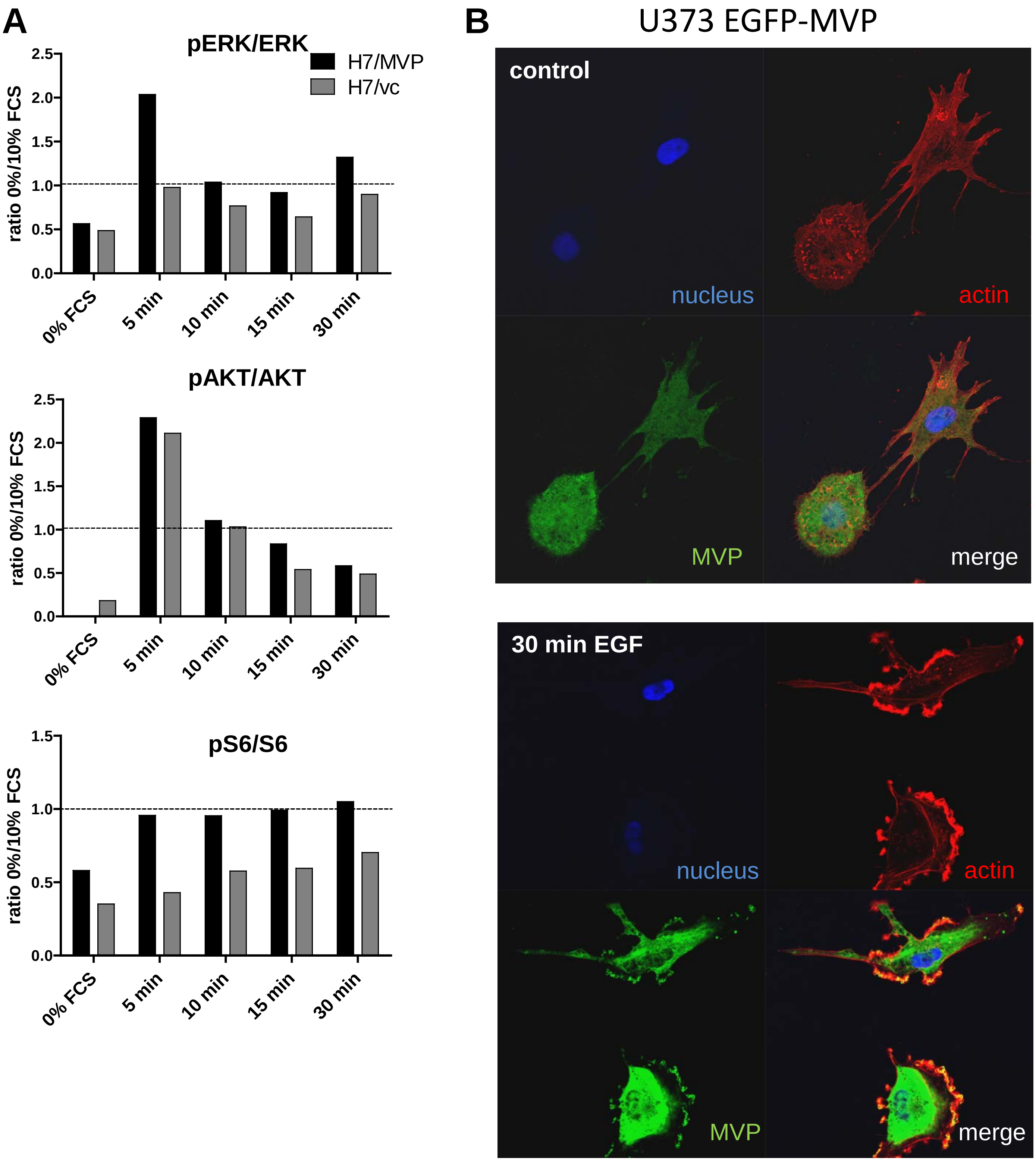

Figure S6: MVP expression and the EGF/EGFR signalling axis in GBM. A Band intensities of Westerns blots shown in Figure 5D were quantified. Ratio of phosphorylated to total proteins was evaluated for starved (0% FCS) and EGF stimulated H7/MVP and H7/vc cells. Values are expressed relatively to controls grown in 10% FCS set as 1. B. Localisation of EGFP-MVP (green) in serum-starved cells (upper panel) as well as after EGF stimulation (lower panel) in U373 cells. The nuclei were counterstained with DAPI (blue) and TRITC-phalloidin was used for the staining of microfilaments (red).
