# Supplementary material for: Major vault protein supports glioblastoma survival and migration by upregulating the EGFR/PI3K signalling axis
Source: Oncotarget. 2013 Sep 1;4(11):1904–18. doi: 10.18632/oncotarget.1264 (PMC3875758; doi:10.18632/oncotarget.1264)
Supplement: Supplementary file 2 [file oncotarget-04-1904-s002.doc]

**Supplementary Material and Methods**

Cloning and adenoviral expression of MVP/EGFP chimeras

For generation of a plasmid constructs that express recombinant enhanced green fluorescence (EGFP)-tagged MVP (pEGFP-MVP), the MVP cDNA was taken out by digestion with *Eco*RI/*Hin*dIII from L-MVP construct and cloned into respective sites of pBS SK(-) (Stratagene). A short fragment encoding a negative-regulating uORF of MVP was removed and the pEGFP-MVP construct generated as published . This construct was digested with *Nhe*I/*Hin*dIII*,* resulting a 3530bp fragment encoding the complete EGFP-MVP chimera and was then inserted into *Xba*I/*Hin*dIII of S-MVP-3 empty pSport6 vector (Invitrogen) backbone . To create a dominant-negative EGFP-tagged MVP-deletion construct (dn pEGFP-MVP), lacking the 7 MVP repeats and the EF-hand calcium-binding domain, the first 1195 bp at the 5´-end of the MVP gene were deleted from pEGFP-MVP by digestion with *Bss*HII and *Bsr*GI, following a fill-in reaction by Klenow enzyme before regulation. The resulting construct was digested with Nhe*I*/*Hin*dIII and inserted into the S-MVP-3 empty pSport6 vector backbone as described before. Both, the pEGFP-MVP and the dn pEGFP-MVP were digested with *Sal*I/*Hin*dIII and fragments with encoded protein chimera were cloned into pShuttle-CMV vector (Stratagene) for generation of recombinant adenovirus with AdEasy system as described . An EGFP adenoviral expression vector was used as control . Virus titres were determined by Adeno-X Rapid Titer Kit (Clontech) and by GFP fluorescence-activated cell sorting (FACS) analyses (FACScalibur; BD Biosciences).

Isolation of RNA and quantitative reverse transcription-polymerase chain reaction (qRT-PCR)

#### Total RNA was extracted from cell cultures using Trizol reagent (Life Technologies, Carlsbad, CA). One µg RNA per sample were reverse transcribed with RevertAid MMLV reverse transcriptase (Thermo Scientific) and random hexamer primers (GE Healthcare). qRT-PCR was performed with Maxima SYBR Green qPCR master mix (Thermo Scientific), as described earlier . For MVP and ß-actin mRNA expression the following primers were used: MVP (sense 5′-TTCTGGATTTGGTGGACGC-3′; antisense 5′-ACTTCTCTCCCTTGACCAC-3′) and ß-actin (sense 5´-GGATGCAGAAGGAGATCACTg-3´; antisense 5´-CGATCCACACGGAGTACTTG -3´). ß-actin served as housekeeping gene and was used for normalization. All reactions were performed in triplicates and repeated independently at least twice.

Gene expression analysis

Gene expression levels of *MVP* in extended collections of normal and malignant brain tissues were extracted from the BioExpress database (GeneLogic) as described . Transcription levels were determined by Affymetrix GeneChip analysis (chip sets HG-U133 A and HG-U133 B, probe set 202180_s_at) in human tissues and human cell lines.

**Supplementary References**

1. Holzmann K, Ambrosch I, Elbling L, Micksche M, Berger W. A small upstream open reading frame causes inhibition of human major vault protein expression from a ubiquitous mRNA splice variant. FEBS Lett. 2001;494:99-104.

2. Steiner E, Holzmann K, Pirker C, Elbling L, Micksche M, Sutterluty H, et al. The major vault protein is responsive to and interferes with interferon-gamma-mediated STAT1 signals. J Cell Sci. 2006;119:459-69.

3. Mauritz I, Westermayer S, Marian B, Erlach N, Grusch M, Holzmann K. Prostaglandin E(2) stimulates progression-related gene expression in early colorectal adenoma cells. Br J Cancer. 2006;94:1718-25.

4. Fischer H, Taylor N, Allerstorfer S, Grusch M, Sonvilla G, Holzmann K, et al. Fibroblast growth factor receptor-mediated signals contribute to the malignant phenotype of non-small cell lung cancer cells: therapeutic implications and synergism with epidermal growth factor receptor inhibition. Mol Cancer Ther. 2008;7:3408-19.

5. Glatt S, Halbauer D, Heindl S, Wernitznig A, Kozina D, Su KC, et al. hGPR87 contributes to viability of human tumor cells. Int J Cancer. 2008;122:2008-16.
